# Experimental Study on the Azimuthal-Transmitting Transducer of Acoustic Logging

**DOI:** 10.3390/s23063247

**Published:** 2023-03-19

**Authors:** Junqiang Lu, Baiyong Men, Xiaohua Che

**Affiliations:** 1 State Key Laboratory of Petroleum Resources and Prospecting, China University of Petroleum (Beijing), Beijing 102249, China; 2 Key Laboratory of Earth Prospecting and Information Technology, Beijing 102249, China

**Keywords:** acoustic logging, azimuth, experiment, transmitting transducer, arc array

## Abstract

Azimuthal acoustic logging can survey the downhole formation more accurately, and the acoustic source is the crucial component of the downhole acoustic logging tool with azimuthal resolution characteristics. To realize downhole azimuthal detection, assembling multiple transmitting piezoelectric vibrators in the circumferential direction is necessary, and the performance of azimuthal-transmitting piezoelectric vibrators needs attention. However, effective heating test and matching methods are not yet developed for downhole multi-azimuth transmitting transducers. Therefore, this paper proposes an experimental method to comprehensively evaluate downhole azimuthal transmitters; furthermore, we analyze the azimuthal-transmitting piezoelectric vibrator parameters. This paper presents a heating test apparatus and studies the admittance and driving responses of the vibrator at different temperatures. The transmitting piezoelectric vibrators showing a good consistency in the heating test were selected, and an underwater acoustic experiment was performed. The main lobe angle of the radiation beam, horizontal directivity, and radiation energy of the azimuthal vibrators and azimuthal subarray are measured. The peak-to-peak amplitude radiated from the azimuthal vibrator and the static capacitance increase with an increase in temperature. The resonant frequency first increases and then decreases slightly with an increase in temperature. After cooling to room temperature, the parameters of the vibrator are consistent with those before heating. Hence, this experimental study can provide a foundation for the design and matching selection of azimuthal-transmitting piezoelectric vibrators.

## 1. Introduction

The demand for azimuthal acoustic logging and three-dimensional acoustic logging is increasingly necessary with the exploration and development of complex oil and gas reservoirs. Acoustic logging with azimuthal resolution in open wells can evaluate the heterogeneity and anisotropy of oil and gas reservoirs. Acoustic logging with azimuthal resolution in cased wells can be used to considerably improve the accuracy of cement bond quality evaluations [1,2].

The traditional acoustic logging transducer primarily contains monopole sound and dipole sound sources. The monopole sound source is symmetrical and has no directivity in the circumferential direction. A sound source without azimuthal resolution can only survey the downhole formation in a two-dimensional space (axial and radial). The dipole sound source has a 180° symmetrical azimuthal characteristic. The sound source has multiple solutions for azimuth identification, and there are certain limitations in determining the orientation of downhole geological bodies [3,4]. The cutoff frequency phenomenon of the borehole flexural wave is used to emit an acoustic wave from a borehole dipole source below the cutoff frequency [5], which only reduces the main frequency of the tool and does not solve the problem of azimuthal measurement.

The research on new technologies and methods has promoted the development of acoustic logging and provided more solutions for solving complex downhole acoustic measurement problems. Hirabayashi developed high-resolution reflector imaging methods using a trial reflector and cross correlation [6]. The wave fields of a monopole sound source applicable to the logging while drilling (LWD) model were investigated, and the propagation mechanisms of collar waves in the LWD models were revealed [7]. The acoustic LWD experimental measurements were conducted to analyze the excitation and propagation of monopole collar waves in the laboratory [8]. Bennett developed 3D slowness time coherence (STC), and the slowness and propagation direction of a reflected wave field can be determined using a simple ray-tracing inversion and 3D STC [9]. Wang et al. developed a new LWD method with a seismoelectric effect and performed seismoelectric LWD measurements in a scaled sandstone borehole with multipole sources (monopole/dipole/quadrupole) [10]. Guan et al. theoretically analyzed the contributions of the poroelastic-wave potentials to seismoelectromagnetic wave fields and calculated the magnetic field using a curl-free electric field [11]. The methodology based on external and embedded sensors is effective in crack detection [12]. Furthermore, data-driven approaches to solving complex downhole measurement problems were proposed [13,14,15]. All these research results are references for improving the downhole measurement in future.

The sound source is a crucial component of a downhole acoustic logging tool with an azimuthal resolution. The fundamental difficulty of the sound source lies in improving the efficiency of azimuthal identification and the strength of useful signals radiated by a sound source. Qiao et al. [16,17] proposed a method of acoustic measurement using a phased arc array sound source in a well and verified its feasibility in evaluating heterogeneous and anisotropic formations using theory, numerical simulation, physical simulation, and field experiments [18,19,20,21].

The installation of multiple piezoelectric vibrators in the circumferential direction is necessary to realize the downhole azimuthal measurement [22,23,24]. The energy, frequency response, and azimuthal resolution of piezoelectric vibrators have a considerable effect on the measurement results. The measurement results will be wrong if the parameters of the piezoelectric vibrators are inconsistent. Simultaneously, the logging tool works in the downhole high-temperature environment, and the effect of different temperatures on the piezoelectric vibrator must also be considered. Experimental tests are an important method to study the performance of transducers [25,26,27,28]. The existing experimental research only considered the underwater acoustic characteristics of the transducer and did not pay attention to the temperature effects on the transducer [16,17,20,24]. Furthermore, effective heating test methods have not been developed for downhole transducers. The available research also did not provide an experimental method to evaluate the consistency of azimuthal piezoelectric vibrators, which may lead to incorrect results when evaluating the formation anisotropy and the orientation of stratigraphic anomalies in the downhole measurement of the tool.

This paper proposes the heating test and matching methods to comprehensively evaluate the downhole azimuthal piezoelectric vibrators. The admittance and driving responses of azimuthal-transmitting piezoelectric vibrators were investigated at various temperatures. Based on the heating test results, the transmitting piezoelectric vibrators showing a good consistency were chosen, and the underwater acoustic experiment was performed. The horizontal directivity, radiation energy, and main lobe angle of the radiation beam of the azimuthal vibrators and subarray are measured, providing a foundation for the design and matching selection of the piezoelectric vibrator for the azimuthal transmitting transducer.

## 2. Structure of the Azimuthal-Transmitting Sonde

### 2.1. Azimuthal Arc Array

**Figure 1:**
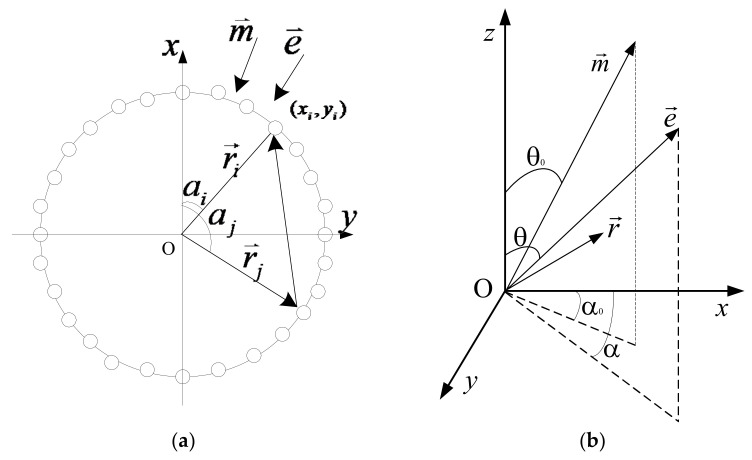
Schematic of acoustic phased arc array and coordinate. (**a**) Coordinates of arc array with uniformly distributed point sources. (**b**) Three-dimensional coordinate.

An azimuthal arc array comprises *N* piezoelectric vibrators on a horizontal plane. Figure 1 shows the schematic of the coordinates used to study the circular arc array. *N* point sources are uniformly arranged on the circumference with radius *a*, and the point sources can be called array elements. The center angle of the circular arc formed by two adjacent array elements is 2*π/N*. The circle lies in the X−Y plane, and the phase difference of the acoustic waves generated by an array element *i* from the acoustic wave incident in any direction (α,θ) and the main maximum direction $\left(\alpha_{0},\theta_{0}\right)$ is expressed as follows:

(1) $$\begin{array}{l} \Delta \phi_{i}=\frac{\omega }{c}a\left[\cos\alpha_{i}(\sin\theta \cos\alpha -\sin\theta_{0}\cos\alpha_{0})+\sin\alpha_{i}(\sin\theta \sin\alpha -\sin\theta_{0}\sin\alpha_{0})\right]\\ =ka\left[\sin\theta \cos(\alpha -\alpha_{i})-\sin\theta_{0}\cos(\alpha_{0}-\alpha_{i})\right] \end{array}$$

where $\alpha_{i}$ is the angle between the vector diameter of element *i* and the positive half axis of the X axis in the X−Y plane, $\alpha_{i}=i2\pi /N$, *c* is the sound velocity, k=ω/c is the wave number, and *a* is the arc array radius. Based on the superposition principle of the sound field, the directivity function of the point source arc array can be expressed as follows:

(2) $$D(\alpha ,\theta ,\alpha_{0},\theta_{0},\omega )=\left|\frac{\sum_{i=1}^{N}A_{i}e^{-j\Delta \phi_{i}}}{\sum_{i=1}^{N}A_{i}}\right|$$

The transmitting time of each element in an azimuthal-transmitting arc array is controlled to adjust the horizontal directivity of the acoustic radiation signal. Using a phase-delayed excitation of adjacent M (M is usually odd and M < N/2) transmitting vibrators, they can radiate sound waves in a certain direction in the horizontal plane. Sound beam scanning in the horizontal plane is achieved by selecting the combination of different azimuthal-transmitting elements in turn.

### 2.2. Azimuthal-Transmitting Acoustic Sonde

**Figure 2:**
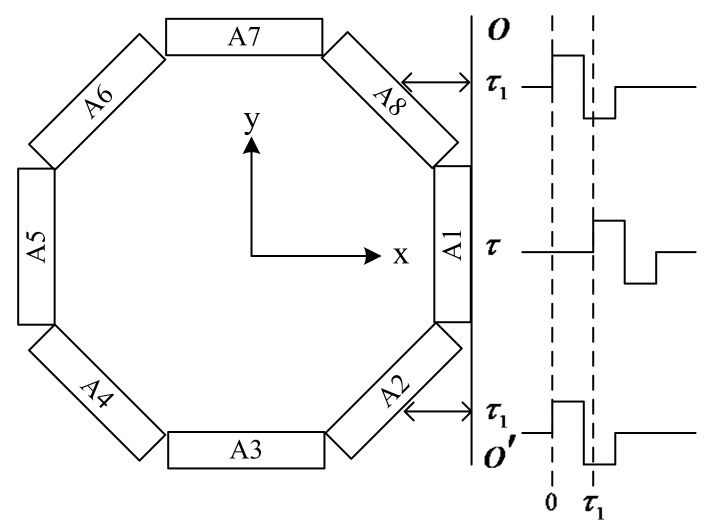
Schematic showing the construction and operational principles of the azimuthal-transmitting arc array.

Figure 2 shows the structure diagram of an azimuthal-transmitting arc array, which is composed of eight elements in actual use. The eight elements (numbered A1–A8) are placed evenly along the circumference, and each element can work independently. Moreover, each element can radiate sound waves in the corresponding direction independently. The energy of the acoustic wave radiated to a certain azimuth can be increased by the phase-delayed excitation of the three adjacent elements, and hence the azimuthal resolution can be improved. For example, the three elements numbered A8, A1, and A2 form a phased transmitting array. Elements A2 and A8 on both sides are excited simultaneously, and the same amplitude excitation signal with a delay time is applied to the central array element A1. Taking adjacent elements, such as A2 and A3, as the center in turn, the three adjacent elements are selected for excitation at different times, the radiated sound beam can be generated in different directions of the circumference, and the circumferential scanning measurement can be achieved.

**Figure 3:**
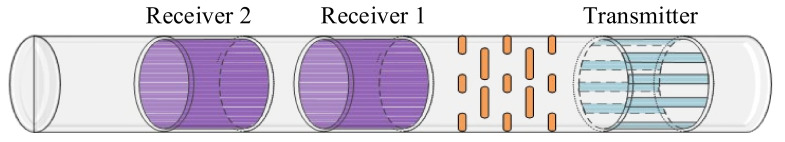
Structure diagram of an acoustic sonde composed of an azimuthal-transmitting arc array.

**Figure 4:**
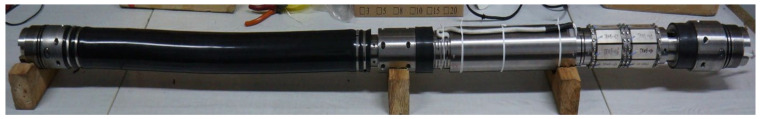
Internal actual picture of an acoustic sonde composed of an azimuthal-transmitting arc array.

An azimuthal acoustic sonde can be fabricated by combining the azimuthal-transmitting arc array with an acoustic receiver. Figure 3 shows its structural diagram. The basic structure of the sound system includes an azimuthal-transmitting arc array and multiple acoustic receivers. Figure 4 shows the internal actual picture of the acoustic logging azimuthal-transmitting sound sonde. Two groups of azimuthal-transmitting arc arrays are used to improve the transmitting energy. Two acoustic receivers are encapsulated in the circular rubber tube.

### 2.3. Transmitting Piezoelectric Vibrator

**Figure 5:**
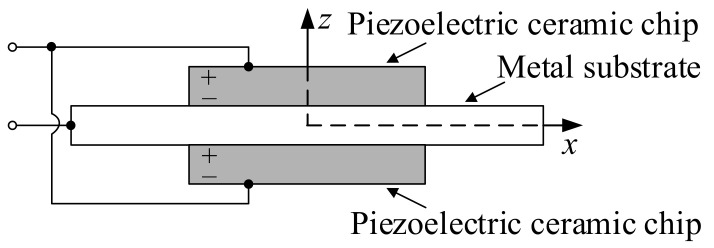
Structural diagram of the transmitting piezoelectric vibrator.

A transmitting piezoelectric vibrator is a three-layered rectangular plate structure composed of a metal substrate and two piezoelectric ceramic chips having identical polarization directions. As shown in Figure 5, the equivalent circuit is a parallel structure. Piezoelectric ceramic chips are bonded to both sides of the metal substrate, and the polarization direction is the thickness direction that is parallel to the *z*-axis. The metal substrate extends beyond the ceramic chips so as to remain connected to the excitation circuit and be supported on the carrier of the arc array. When the excitation signal is applied, the ceramic chip on one side will stretch, whereas that on the other side will shrink, and hence the piezoelectric vibrator will generate a bending vibration and radiate sound waves.

## 3. Experimental Measurement of Transducers

Consistency in the performance of elements in an arc array is the technical index that needs to be focused on in an azimuthal acoustic logging tool. When fabricating the transmitting arc array, a large number of piezoelectric vibrators in the laboratory often need to be tested. Moreover, only those vibrators showing consistently good performances need to be selected to form the azimuthal transmitter. The important indicators for the performance consistency evaluation of piezoelectric vibrators include the admittance, temperature, and transmitting response, and they are interrelated. A good consistency of the admittance is the prerequisite to meeting the other two indicators.

### 3.1. Heating Test

The admittance characteristics of piezoelectric vibrators in the free state in silicon oil are measured at room temperature. Piezoelectric vibrators with good admittance consistency are preliminarily selected based on the principle that the resonant frequency and the static capacitance of piezoelectric vibrators are as close or equal as possible.

**Figure 6:**
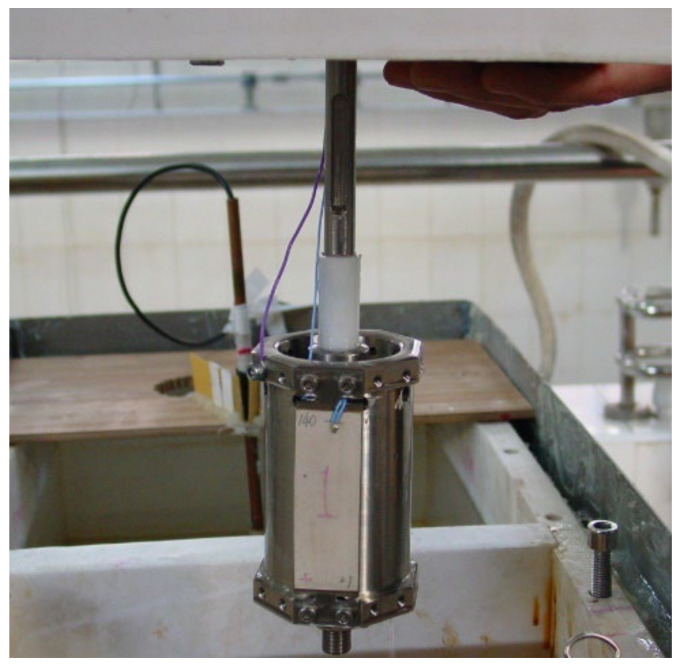
Physical diagram of the experimental heating device of transmitting piezoelectric vibrators.

**Figure 7:**
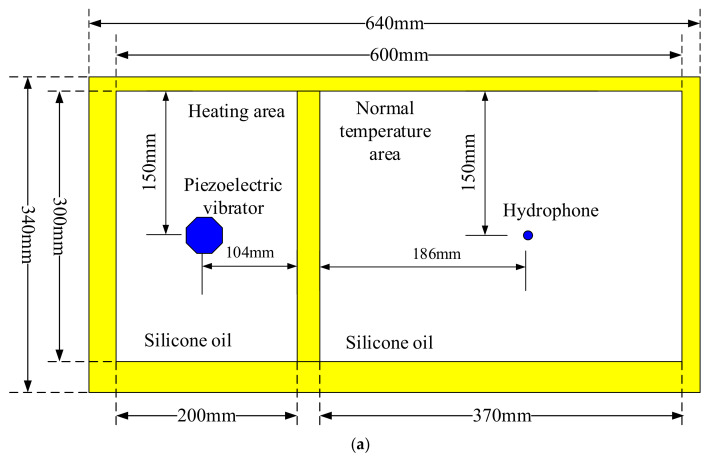

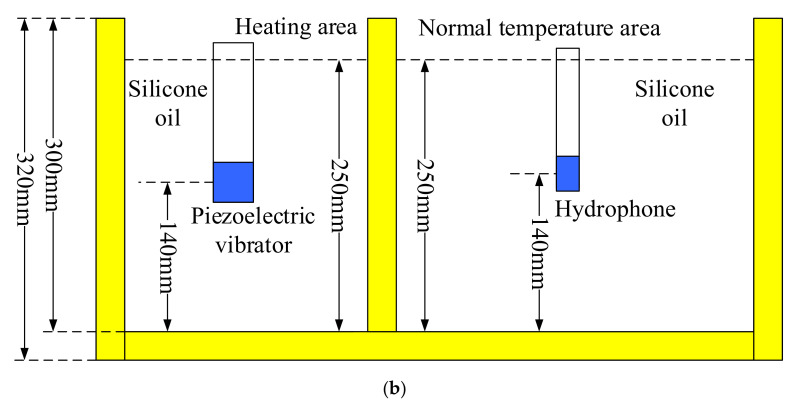
Schematic of the experimental heating device of the transmitting piezoelectric vibrator. (**a**) Vertical view and (**b**) lateral view.

The parameter consistency of the preliminarily selected piezoelectric vibrators is tested at various temperatures. Figure 6 and Figure 7 show the physical and schematic diagrams, respectively, of the experimental heating device. The test areas include the heating and normal temperature areas, which are filled with silicone oil. The piezoelectric vibrator to be tested is placed in the heating area, and the receiver is placed in the normal temperature area. The temperature control device adjusts the temperature of the silicone oil in the heating area. The admittance and excitation responses of the piezoelectric vibrators are analyzed at different temperatures. During the test, the transmitting piezoelectric vibrator is clamped to the arc array carrier; furthermore, the entire arc array is immersed in silicone oil. A transmitting piezoelectric vibrator is continuously excited by a high-voltage pulse signal, and a receiver acquires the waveform in the normal temperature area when its excitation response is measured. The piezoelectric vibrators with good temperature resistance are selected based on the principle that the resonant frequency, static capacitance, and excitation response are equal or similar, and the underwater acoustic response tests of the preferred vibrators are conducted in water.

### 3.2. Underwater Acoustic Response Test

**Figure 8:**
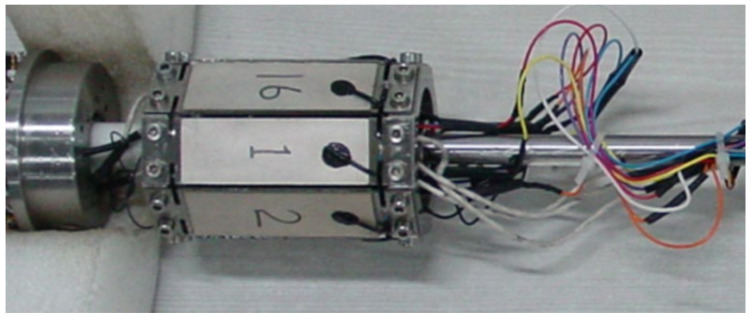
Physical installation diagram of the underwater acoustic response test of transmitting piezoelectric vibrators.

During the experimental measurement, eight transmitting piezoelectric vibrators were installed in the test device to form an azimuthal-transmitting sound system. The mounting mode of piezoelectric vibrators is similar to the actual tool, and they are uniformly arranged in a circumferential direction. Figure 8 shows the physical installation diagram of the piezoelectric vibrators. When the underwater acoustic response of the transmitting piezoelectric vibrator is tested, the hydrophone is fixed, the transmitting vibrator is rotated around the central axis of the azimuthal-transmitting sound system, and the measurement is conducted during the rotation. The transmitting piezoelectric vibrator and the hydrophone are placed on a similar horizontal plane by adjusting the height of the sound system. The distance between the transmitting sound system and the hydrophone is 2000 mm, thereby meeting the free far-field conditions. Additionally, the positions of both are as far away as possible from the reflective interfaces during the measurement process, which can reduce the boundary effects.

**Figure 9:**
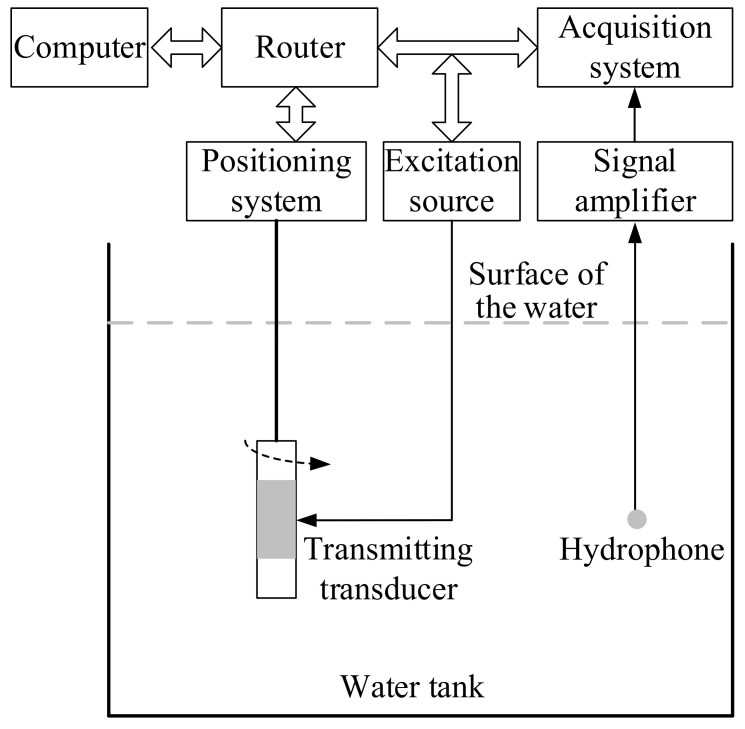
Schematic diagram of the underwater acoustic response experimental device for transmitting piezoelectric vibrators.

The underwater acoustic measurement of azimuthal piezoelectric vibrators is performed in a non-anechoic experimental pool with a size of 5 m × 5 m × 4 m. Water density and sound velocity are 1.0 g/cm^3^ and 1500 m/s, respectively. As shown in Figure 9, the experimental measurement system mainly comprises a computer, a positioning system, a multichannel high-power signal excitation source, a multichannel acquisition system, a controllable-gain signal amplifier, a standard B&K 8103 hydrophone, and a digital oscilloscope. The positioning system is primarily used for precisely controlling the motion of the hydrophone and the azimuthal vibrators and for adjusting the distance between the transmitter and the receiver. It has two working heads (HEAD1 and HEAD2), each of which has a translational degree of freedom in the X, Y, and Z directions and a rotational degree of freedom in the C direction. The multichannel high-power excitation source can output 32 independent channels of high-voltage pulse signals. The data acquisition system contains 16 independent channels with an acquisition accuracy of 16 bits as well as a maximum sampling rate of 1 MHz and a maximum sampling point of 8192. The hydrophone is installed at the bottom end of the working head HEAD2; furthermore, it has a flat receiving sensitivity response and omnidirectional directivity in the frequency range of 0.1 Hz–180 kHz. The controllable-gain signal amplifier is battery-powered with amplification and filtering functions and can be adjusted in the range of 0–80 dB.

## 4. Analysis of Experimental Results

### 4.1. Analysis of Heating Test Results

**Figure 10:**
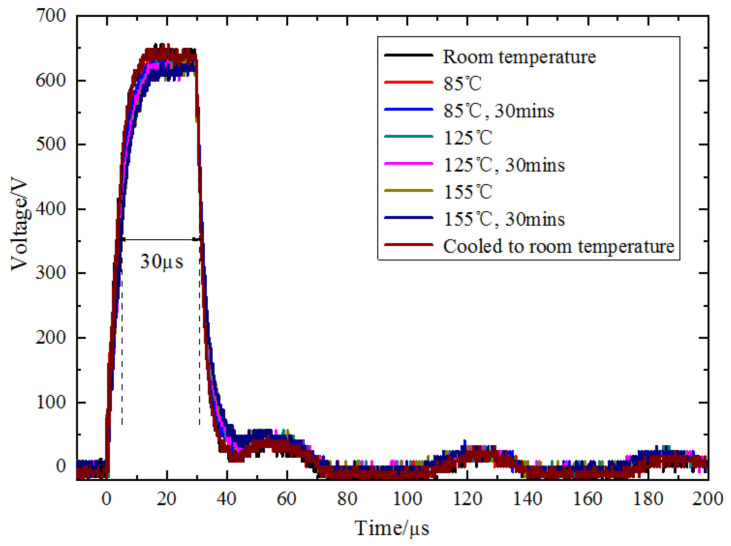
Excitation pulse signals of a piezoelectric vibrator at different temperatures.

**Figure 11:**
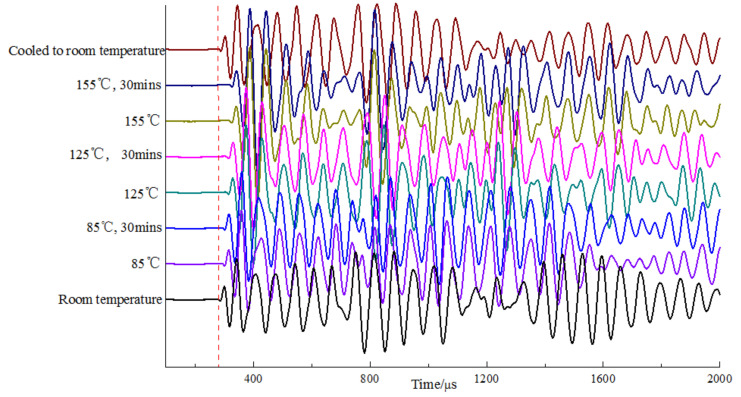
Time domain waveforms received by the receiver in the normal temperature area, when a piezoelectric vibrator transmits pulse acoustic waves at different temperatures.

The excitation pulse signals and admittance characteristics of the transmitting piezoelectric vibrators at various temperatures (room temperature; 85 °C and 85 °C for 30 min; 125 °C and 125 °C for 30 min; 155 °C and 155 °C for 30 min; and cooling to room temperature) are measured, and the time-domain waveforms received by the receiver are also acquired. Figure 10 shows the excitation pulse signals of a piezoelectric vibrator at different temperatures. With an increase in the temperature, the rising edge of the pulse signal gradually decreases and the pulse amplitude slightly decreases. The relative decrease in the pulse amplitude is <3.7%, whereas the pulse width remains the same. The piezoelectric vibrator emits pulsed acoustic waves at different temperatures. Figure 11 shows the time-domain waveforms received by the receiver in the normal temperature area. The arrival time of the first wave in the time-domain waveform gradually increases with an increase in the temperature. The peak-to-peak value of the time-domain waveform in the first 1.5 quasi-periods is utilized to measure the transmitting performance of the piezoelectric vibrator, and the values at different temperatures are presented in Table 1. The peak-to-peak values of the received waveforms generally increase gradually with an increase in the temperature. At 155 °C for 30 min, the peak-to-peak value increased by 42.61% compared with that at room temperature. The excitation pulse varies slightly with an increase in the temperature; however, the amplitude of the receiving waveform shows considerable changes and is mainly due to a reduction in the energy radiation efficiency of the transmitting transducer when the temperature increases. When the temperature decreases to room temperature, the peak-to-peak value of the received waveform is slightly higher than that before heating, with a relative increase of about 5.0%.

**Figure 12:**
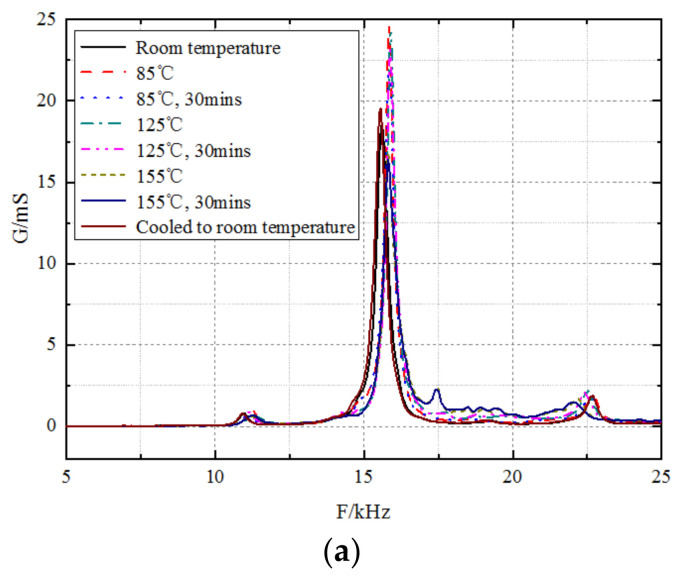

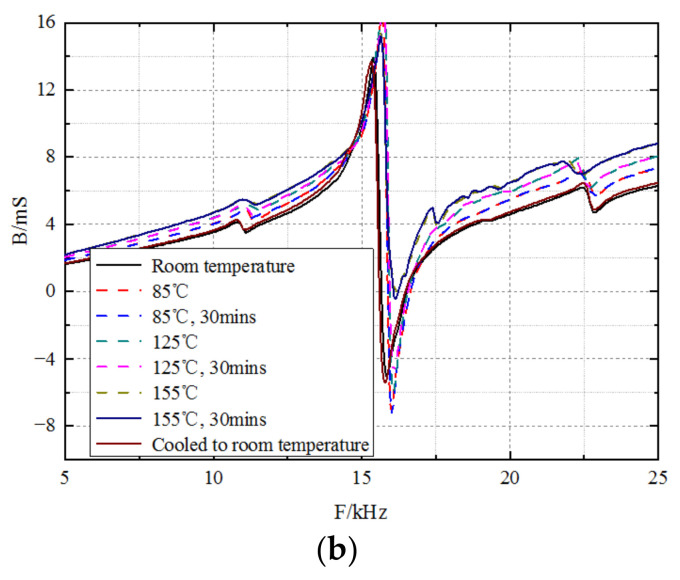
Admittance curves of a piezoelectric vibrator at different temperatures. Curves of the (**a**) conductance and frequency and (**b**) susceptance and frequency.

Figure 12 shows the admittance–frequency curves of a piezoelectric vibrator at different temperatures, where (a) is the conductance–frequency curves and (b) is the susceptance–frequency curves. Table 1 presents the resonant frequency, static capacitance, and maximum conductance of the piezoelectric vibrator at various temperatures. The resonant frequency of the piezoelectric vibrator first increases with an increase in temperature and then decreases slightly. However, the resonant frequency at high temperatures is larger than that at normal temperatures, and the relative increase at a high temperature of 155 °C is 1.35%. The static capacitance increases with an increase in temperature, and the relative increase at 155 °C is 41.12%. The resonant frequency, static capacitance, and maximum conductance of the piezoelectric vibrator are approximately the same as those before heating when the temperature is reduced to normal temperature. The experimental results show that the transmitting piezoelectric vibrators can work stably at various temperatures and can be recovered when they are cooled to room temperature, thereby meeting the requirements of the downhole high-temperature tool. The heating test results of other piezoelectric vibrators are similar to these results.

### 4.2. Analysis of Sound Field Test Results

**Figure 13:**
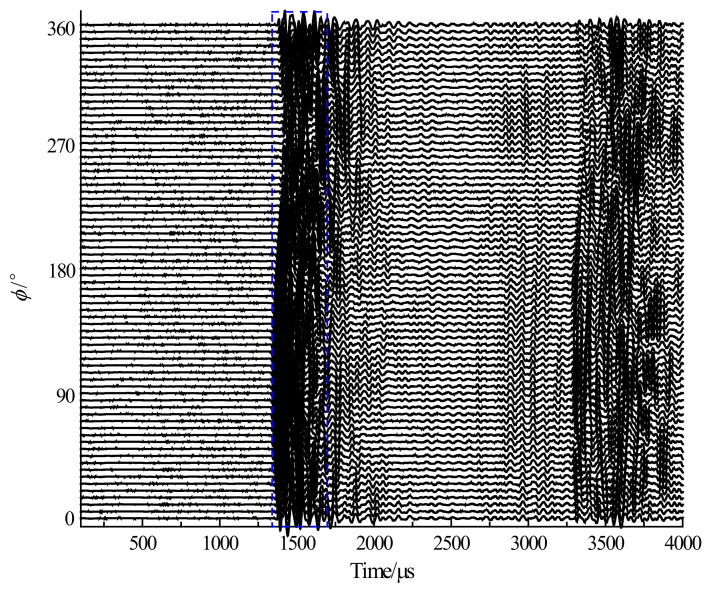
Time-domain waveforms of the sound pressure received by the hydrophone when a single piezoelectric vibrator works.

Figure 13 shows the sound pressure waveforms of a single transmitting piezoelectric vibrator. The arc array rotates once every 5° and emits an acoustic pulse, and the hydrophone receives the time-domain waveform.

**Figure 14:**
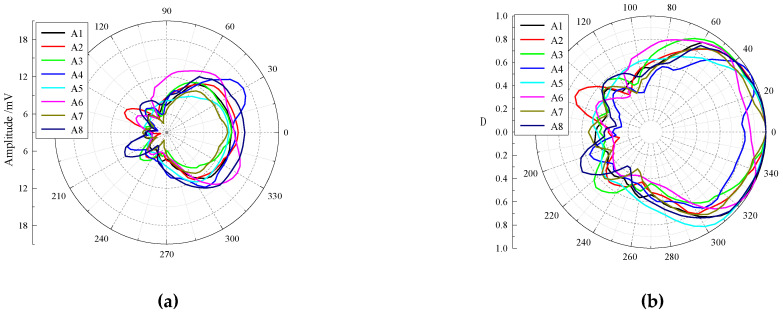
Horizontal directivity of azimuthal-transmitting piezoelectric vibrators. (**a**) Non-normalized horizontal directivity and (**b**) normalized horizontal directivity.

The peak-to-peak values of the time-domain waveforms are estimated in the time window (navy blue lines)of 1300–1550 μs, and the horizontal directivities of the transmitting piezoelectric vibrators are obtained, as shown in Figure 14. When a single piezoelectric vibrator works, the main lobe direction of the radiation beam is basically along the 0° direction, and the radiation beam is generally symmetrical. However, the main lobes of the piezoelectric vibrators A4 and A6 are relatively flat in the 0° direction. A series of side lobes are found in other directions except for the main lobe, but the radiation energy of the side lobes is weak. A good degree of consistency is exhibited by the radiation energy and the −3 dB angular width in the main lobe direction of each transmitting piezoelectric vibrator, but a certain degree of discreteness is also observed. Among them, the peak-to-peak value in the main lobe direction of the piezoelectric vibrator A8 is the largest, whereas the −3 dB angular width in the main lobe direction of the piezoelectric vibrator A4 is the smallest. Table 2 presents the experimental test results for each piezoelectric vibrator.

The three adjacent transmitting vibrators can form a transmitting phased array. Each element is excited by the pulse excitation signals with a phase difference in the transmitting phased array, which can improve the acoustic energy radiated by the transmitter and reduce the main lobe angle of the radiated acoustic beam. The peak voltage of the pulse excitation signal and the pulse width are 500 V and 30 μs, respectively. The directivity of the phased array is measured when the excitation signals of adjacent elements have no phase difference and have a phase difference.

**Figure 15:**
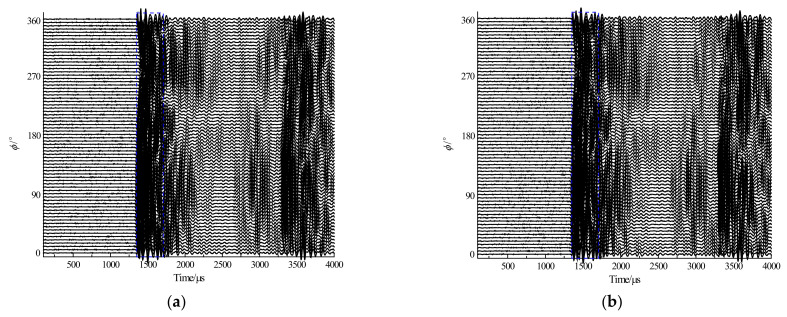
Time-domain waveforms received by the hydrophone when each element in the subarray is applied with an excitation signal, the navy lines represent the time window for extracting waveform amplitude. (**a**) Waveforms with no phase delay of excitation signals and (**b**) waveforms with a phase delay of excitation signals.

**Figure 16:**
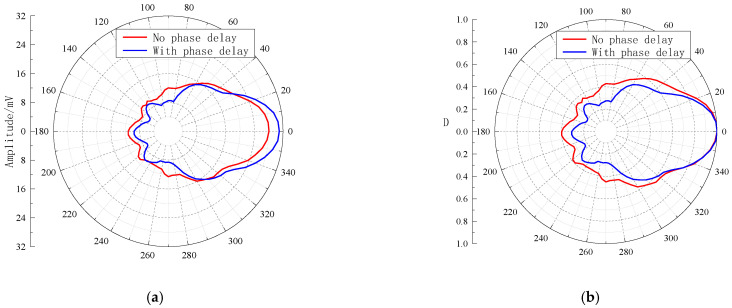
Horizontal directivity curves of the acoustic field radiated by the transmitting phased array. (**a**) Non-normalized curves and (**b**) normalized curves.

Figure 15 shows the time-domain waveforms received by the hydrophone when each element in the subarray with A1 as the center is applied with an excitation signal, where (a) and (b) show the waveforms without and with a phase delay of the excitation signals, respectively. As shown in Figure 16, the horizontal directivity curves of the acoustic field radiated by the transmitting phased array can be obtained, where (a) and (b) are the non-normalized and normalized curves, respectively. The main lobe of the radiated sound beam is basically near the 0° direction, and the radiated sound beam is symmetrically distributed in the main lobe direction. When the excitation signals have no phase delay, the peak-to-peak amplitude in the main maximum direction of the radiated sound field is 28.04 mV, and the −3 dB angular width of the directivity is 64°. Moreover, when the excitation signals have a phase delay, the peak-to-peak amplitude in the main maximum direction of the radiated sound field is 30.93 mV, and the −3 dB angular width of the directivity is 55°. Furthermore, when the transmitting subarray works in phased mode, the energy of the radiated sound beam is considerably increased, whereas the −3 dB angular width of the main lobe is remarkably decreased. This enhances the azimuthal detection capability and improves the signal-to-noise ratio of the useful signal.

## 5. Conclusions

An azimuthal-transmitting arc array can obtain the circumferential scanning radiation measurement, solve the problem that the transmitting transducer of a traditional acoustic logging tool cannot estimate the downhole azimuthal measurement, and increase the radiation energy of the transmitting transducer. This can enhance the amplitude of the received signals and improve the accuracy of measurement.

A heating test system for estimating the downhole measurements at various temperatures was designed, and the characteristics of the transmitting piezoelectric vibrators at different temperatures were analyzed. Based on the optimal selection of transducers using the heating test, the underwater acoustic test was performed, which can better simulate the actual downhole measurement environment and is conducive to improving the stability and accuracy of downhole tools.

With an increase in temperature, the peak-to-peak amplitudes of acoustic wave signals radiated by transmitting piezoelectric vibrators and the static capacitances of the vibrators increase, whereas the resonant frequency increases first and then decreases slightly. At 155 °C for 30 min, the peak-to-peak amplitude of the receiving waveform is 42.61% higher compared with that at room temperature; moreover, the relative increase in static capacitance is 41.12% and the resonant frequency is 1.35%. After cooling to room temperature, the peak-to-peak amplitude of the receiving waveform, the resonant frequency, and the static capacitance of the piezoelectric vibrator are essentially similar to those before heating. The experiment shows that the transmitting piezoelectric vibrators work stably at various temperatures and can be recovered when they are cooled to room temperature, thereby meeting the requirements of a downhole high-temperature tool.

The main lobe of the radiation beam of each transmitting piezoelectric vibrator shows that the horizontal directivities of the vibrators that were selected through the heating test are consistent. A single azimuthal piezoelectric vibrator shows obvious azimuthal radiation characteristics. When the phased subarray is used, the energy of the acoustic beam radiated by the transmitter is considerably increased and the −3 dB angle width of the main lobe of the acoustic beam is significantly decreased. This is conducive to enhancing the azimuthal resolution of downhole tools and improving the signal-to-noise ratio of useful signals.

## Figures and Tables

**Table 1:** Experimental test results of a piezoelectric vibrator at different temperatures.

Temperature	Peak-to-Peak Value		Resonant Frequency		Static Capacitance		Maximum Conductance	
	Measured Value (mV)	Relative Variation (%)	Measured Value (kHz)	Relative Variation (%)	Measured Value (nF)	Relative Variation (%)	Measured Value (mS)	Relative Variation (%)
Room temperature	37.20	0	15.60	0	45.91	0	18.50	0
85 °C	43.24	16.24	15.85	1.60	54.42	18.54	24.64	33.19
85 °C, 30 min	45.37	21.96	15.85	1.60	54.03	17.69	22.44	21.30
125 °C	48.69	30.89	15.90	1.92	59.95	30.58	24.44	32.11
125 °C, 30 min	49.61	33.36	15.88	1.79	59.66	29.95	23.11	24.92
155 °C	52.44	40.97	15.81	1.35	64.79	41.12	16.72	−9.62
155 °C, 30 min	53.05	42.61	15.81	1.35	64.39	40.25	16.37	−11.51
Cooled to room temperature	39.05	4.97	15.55	−0.32	47.34	3.11	19.52	5.51

**Table 2:** The transmitting piezoelectric vibrator is directly opposite to the hydrophone; and the peak-to-peak amplitude, dominant frequency, and peak-to-peak sound pressure of the time-domain waveform are measured. The main lobe width of −3 dB is obtained by directivity measurement. The source distance between the transmitter and receiver is 2000 mm.

No. of Piezoelectric Vibrators	Peak-to-Peak Amplitude (mV)	Dominant Frequency (kHz)	Peak-to-Peak Sound Pressure (Pa)	Main Lobe Width of −3 dB (°)
A1	13.29	14.65	730.34	142
A2	14.47	14.89	795.19	140
A3	13.39	14.16	735.84	145
A4	14.01	14.40	769.91	120
A5	13.02	14.65	715.50	146
A6	13.88	14.40	762.76	151
A7	12.82	14.16	704.51	142
A8	15.55	15.14	832.0	144
Average value	13.82	14.56	759.47	141

## Data Availability

The data presented in this study are available on request from the corresponding author.
